# Efficacy and safety of lung-protective ventilation in neurosurgery: a systematic review and meta-analysis of randomized controlled clinical trials

**DOI:** 10.3389/fmed.2026.1803798

**Published:** 2026-04-23

**Authors:** Yan Liu, Qian Cao, Qian-Yun Pang, Hongliang Liu

**Affiliations:** 1 Zhuhai People's Hospital, Zhuhai, China; 2 Dazhou Dachuan District People's Hospital, Dazhou, China; 3 Chongqing University Cancer Hospital, Chongqing, China

**Keywords:** intracranial pressure, lung-protective ventilation, neurosurgery, positive end expiratory pressure, postoperative pulmonary complication, safety

## Abstract

**Methods:**

We systematically searched the PubMed, Embase, the Cochrane Library, and WANFANG databases (2000–2025) for randomized controlled trials (RCTs) comparing lung-protective ventilation (LPV) with conventional ventilation in craniotomy patients. The primary outcome was the incidence of overall postoperative pulmonary complications (PPCs). The secondary outcomes included intracranial pressure (ICP), pulmonary infection, atelectasis, oxygenation index, and lung compliance. All analyses were performed using Review Manager 5.2.

**Results:**

Seven RCTs involving 523 patients were included in the study. Compared to conventional ventilation, LPV significantly reduced the risk of overall PPCs (OR 0.30, 95% CI: 0.18–0.48, *p* < 0.00001, *I*^2^ = 0%), without increasing the optic nerve sheath diameter (ONSD)—a surrogate measure for ICP either before dual opening (MD: -0.01, 95%CI: −0.04–0.02, *p* = 0.45, *I*^2^ = 0%) or at the end of surgery (MD: −0.04, 95%CI: −0.10–0.02, *p* = 0.21, *I*^2^ = 0%). LPV significantly improved lung compliance (MD: 1.81, 95%CI: 0.79–2.84, *p* = 0.0005, *I*^2^ = 81%) and oxygenation (MD: 40.28 mmHg, 95%CI: 24.93–56.03, *p* < 0.00001, *I*^2^ = 59%) at the end of surgery, while also decreasing the risk of postoperative pulmonary infection (OR: 0.36, 95%CI: 0.22–0.59, *p* < 0.0001, *I*^2^ = 12%) and atelectasis (OR: OR: 0.15, 95%CI: 0.08–0.30, *p* < 0.00001, *I*^2^ = 14%).

**Conclusion:**

This meta-analysis demonstrates that LPV effectively reduces PPCs (moderate-quality evidence) and does not elevate ICP (low- to moderate-quality evidence). Although conclusions regarding ICP are based on surrogate measures, further large-scale RCTs with standardized measures of invasive ICP and consistent definitions of PPCs and LPV are required to validate our findings.

## 1 Introduction

The incidence of postoperative pulmonary complications (PPCs) in neurosurgery can be as high as 25% (1), which negatively impacts clinical outcomes. Intraoperative lung-protective ventilation (LPV) strategies are recommended to reduce PPCs by improving intraoperative oxygenation and lung compliance. These strategies involve the use of a low tidal volume (Vt, ≤8 mL/kg, predicted body weight) and positive end-expiratory pressure (PEEP, typically ≥5cmH2O or at an optimal level), with or without a recruitment maneuver (RM) (2). Recent studies have reported conflicting findings regarding the effects of LPV on PPCs in neurosurgery (3–5). Thus, the efficacy of LPV in reducing PPCs among neurosurgical patients remains a topic of debate.

However, LPV is rarely used or applied cautiously in neurosurgery due to theoretical concerns: low Vt may induce hypercapnia, while higher PEEP could potentially increase intracranial pressure (ICP) and reduce cerebral perfusion (3). Since PEEP is a key component in LPV, it is crucial to consider whether LPV causes ICP elevation when using it to reduce PPCs in neurosurgery. This is particularly important for patients with preexisting pathology (such as brain tumors or injuries). Therefore, the safety of LPV in neurosurgery remains a major concern.

To elucidate the safety and efficacy of intraoperative LPV in neurosurgery, we conducted this systematic review and meta-analysis. It aims to determine the effects of LPV on ICP and PPCs, thereby providing evidence-based guidance for the application of LPV in neurosurgical procedures.

## 2 Methods

This meta-analysis was conducted in accordance with the Preferred Reporting Items for Systematic Reviews and Meta-analyses (PRISMA) guidelines and was registered in PROSPERO (registration no: CRD420251069282).

### 2.1 Literature search and outcomes

The databases PubMed, Cochrane Library, Embase, and WANFANG were searched from January 2000 to April 2025. Search terms included: positive end-expiratory pressure (PEEP), protective lung ventilation, neurosurgery, craniotomy, cerebral surgery, and clinical trials. Detailed search strategies for each database are provided in Supplementary File 1. The references of the included articles were manually screened to identify potentially eligible trials. No restrictions were applied to the publication language. During the literature search, the screening of titles and abstracts, along with full-text assessments, was performed by two independent reviewers with clinical expertise in this field, and a customized data management table was developed to systematically record the study screening process. The primary outcome was postoperative pulmonary complications (PPCs). Secondary outcomes included intracranial pressure (ICP), pulmonary infection, atelectasis, oxygenation index, and lung compliance.

### 2.2 Inclusion and exclusion criteria

The inclusion criteria were as follows: (1) randomized controlled trials comparing conventional ventilation and lung-protective ventilation during craniotomy, (2) adult patients, and (3) full-text publications with sufficient data for at least one of the outcomes (PPCs or ICP). The exclusion criteria included the following: (1) clinical trials lacking adequate data on PPCs, ICP, or oxygenation; (2) studies focusing exclusively on spinal surgery; (3) trials comparing different protective ventilation strategies without conventional ventilation as a control group; and (4) abstracts, editorials, or case reports.

### 2.3 Data extraction and quality assessment

Data were extracted independently by two authors in accordance with the inclusion and exclusion criteria. Any discrepancies were resolved through discussion and consensus among all authors. When data were presented as medians, they were converted to means (standard deviation, SD) using the method described by Wan et al. (6). Data from graphs were extracted using Plot-Digitizer software.

The risk of bias was assessed based on the following domains, as recommended by the Cochrane Collaboration: “adequate sequence generation,” “allocation concealment,” “blinding,” “incomplete outcome data addressed,” “free of selective reporting,” and “free of other bias.” The methodological quality of the included studies was evaluated using the Jadad scoring system by the same two reviewers who had experience in methodological quality assessment. This system considers sample size calculation, generation of the allocation sequence, allocation concealment, methods of randomization, blinding, and documentation of protocol deviations, withdrawals, and dropouts. Any discrepancies were resolved through discussion and consensus among all authors. Studies with a Jadad score below 3 were excluded from this analysis. The Grading of Recommendations, Assessment, Development, and Evaluation (GRADE) methodology was used to assess the quality of the evidence for each outcome.

### 2.4 Statistical analysis

Meta-analysis was performed using Review Manager version 5.2 for Windows (the Cochrane Collaboration, Oxford, UK). For continuous data, the effect sizes were expressed as mean differences (MDs) for ONSD, OI, or lung compliance with 95% confidence intervals (CIs). For dichotomous data, the effect sizes were presented as odds ratios (ORs) with 95% CIs. The *I*^2^ value was used to assess heterogeneity. In cases of heterogeneity (*I*^2^ ≥ 50%), a random-effects model was employed; a subgroup analysis was conducted to explore potential sources of heterogeneity when necessary; in cases of homogeneity (*I*^2^ < 50%), a fixed-effects model was employed. A sensitivity analysis was conducted to evaluate the robustness of the findings. Publication bias was assessed using a funnel plot.

Trial sequential analysis (TSA) was conducted using TSA 0.9.5.10 Beta software, and the required information size (RIS) for each outcome was estimated with a type I error of 0.05 and a type II error of 0.20 in accordance with the TSA manual.

## 3 Results

### 3.1 Characteristics of the included trials

**Figure 1:**
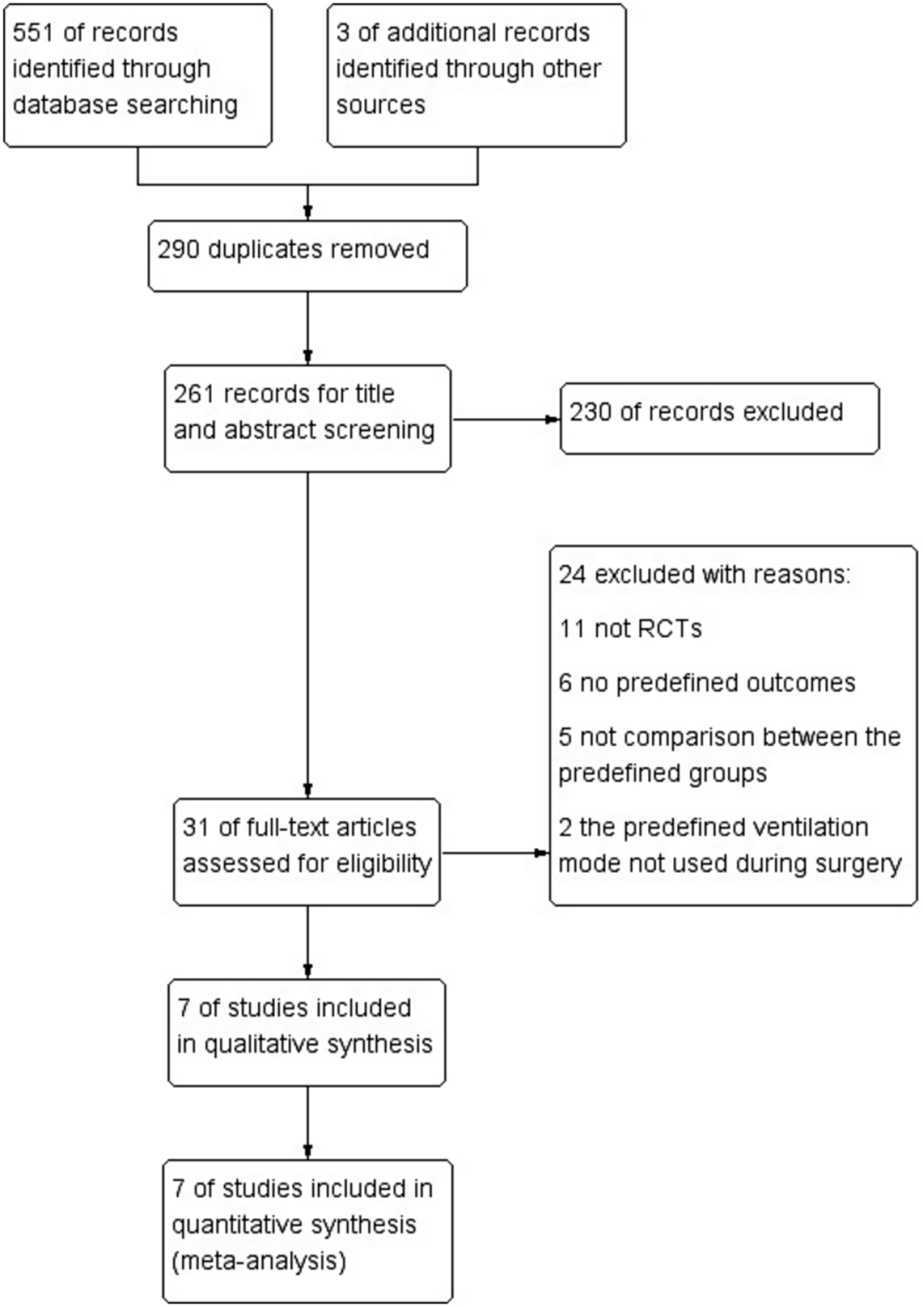
Flowchart of study selection.

**Figure 2:**
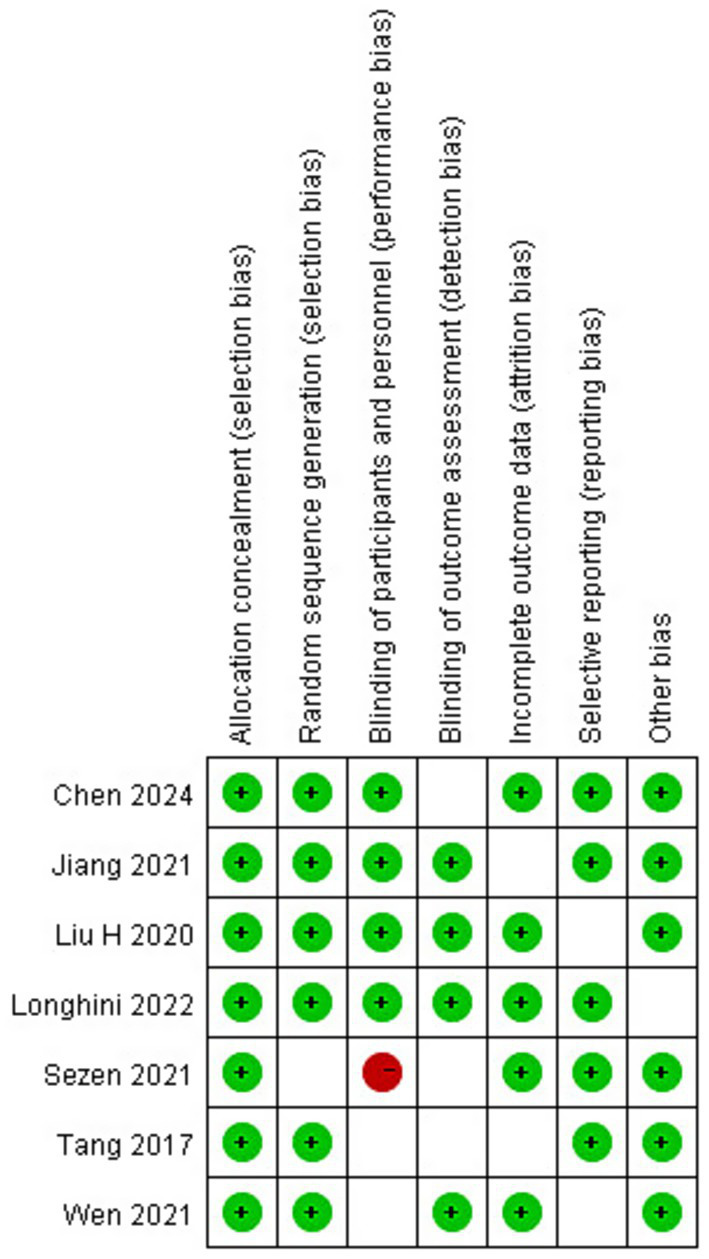
Risk of bias summary. Green indicates a low risk of bias, red indicates a high risk of bias, and white indicates an unclear risk of bias.

A total of 551 articles were identified through the search terms: 214 from PubMed, 69 from Embase, 234 from the Cochrane Library, 31 from WANFANG, and 3 from other sources. After removing duplicates, 261 articles remained. Following title and abstract screening, 230 articles were excluded, leaving 31 full-text articles for the eligibility assessment. Of these, 24 articles were excluded, and 7 RCTs involving 523 patients were included for quality evaluation and qualitative analysis (4, 5, 7–11). Among the seven included studies, four RCTs evaluated patients undergoing elective tumor resection (4, 8, 10, 11), while three other studies assessed patients with traumatic brain injury (5, 7, 9). Four studies used a PEEP of 5 cmH_2_O (5, 7, 9, 11), two studies used a PEEP of 5 cmH_2_O plus a recruitment maneuver (4, 9), and the other two studies adopted an individualized PEEP strategy (5, 8). Three studies used the optic nerve sheath diameter to indirectly assess intracranial pressure (ICP) (5, 9, 11), and one study directly monitored invasive ICP (7). All included studies had a Jadad score above 3. The detailed characteristics of the included studies are presented in Table 1. The study selection process is illustrated in Figure 1, and the risk of bias assessment is shown in Figure 2.

**Table 1:** Characteristics of the included studies.

Author and year	Count-ry	Surgical population	Surgery	Groups and ventilation interventions	Sample size	Outcomes	Jadad score
Longhini et al. (2021) (3)	Italy	Elective tumor	Neurosurgery	Control: Vt 10 mL/kg LPV: Vt6ml/kg + PEEP5cmH2O + RM	*n* = 30 *n* = 30	PPCs, OI	7
Chen et al. (2024) (4)	China	TBI	Craniocerebral surgery	Control: Vt6ml/kg LPV: Vt6ml/kg + PEEP5cmH2O LPV: Vt6ml/kg + PEEP (individualized)	*n* = 30 *n* = 31 *n* = 28	ICP (ONSD), PPCs, OI, lung compliance	7
Wen et al. (2021) (5)	China	TBI	Intracranial evacuation of hematoma	Control: Vt10ml/kg LPV: Vt6-8 ml/kg + PEEP5cmH2O	*n* = 45 *n* = 45	ICP (invasive), PPCs, OI	4
Liu et al. (2020) (8)	China	Elective tumor	Supratentorial tumor resection	Control: Vt8ml/kg LPV: Vt6ml/kg + PEEP (individualized)	*n* = 40 *n* = 40	OI, atelectasis	7
Jiang et al. (2021) (9)	China	TBI	Intracranial evacuation of hematoma	Control: Vt10 ml/kg LPV: Vt8 ml/kg + PEEP5cmH2O LPV: Vt8 ml/kg + PEEP5cmH2O + RM	*n* = 25 *n* = 28 *n* = 26	ICP (ONSD), PPCs, OI, lung compliance	7
Tang et al. (2017) (10)	China	Elective tumor	Craniocerebral surgery	Control: Vt12ml/kg LPV: Vt6ml/kg + PEEP10cmH2O	*n* = 27 *n* = 28	OI, lung compliance	5
Sezen et al. (2021) (11)	Turkey	Elective tumor	Craniotomy	Control: Vt10 ml/kg LPV: Vt8 ml/kg + PEEP 5 cmH2O	*n* = 30 *n* = 30	ICP (ONSD)	4

TBI: traumatic brain injury; LPV: lung-protective ventilation; Vt: tidal volume; PEEP: positive end-expiratory pressure; RM: recruitment maneuver; PPCs: postoperative pulmonary complications; OI: oxygenation index; ONSD: optic nerve sheath diameter; ICP: intracranial pressure.

### 3.2 Meta-analysis

Chen et al. (5) compared conventional ventilation with protective lung ventilation (PEEP 5 cmH2O) or with individualized PEEP. Jiang et al. (9) compared conventional ventilation with protective lung ventilation (PEEP 5cmH2O) or protective lung ventilation (PEEP 5 cmH2O + recruitment maneuvers), and these were split into two substudies in the forest plots for all the following outcomes.

### 3.3 Overall PPCs

The definitions and timing of PPCs in the included studies are provided in the Supplementary Table 1.

**Figure 3:**
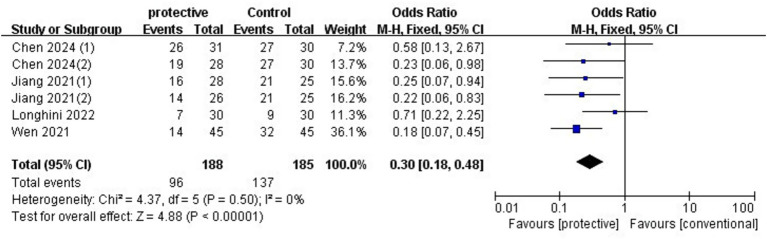
Forest plot of overall PPCs comparing lung ventilation and conventional ventilation.

Data from four studies (*n* = 318) (4, 5, 7, 9) showed that protective lung ventilation significantly reduced PPCs (OR 0.30, 95% CI: 0.18–0.48, *p* < 0.00001, *I*^2^ = 0%) (Figure 3). No publication bias was evident in the funnel plot, and sensitivity analysis revealed that the result was robust even when omitting any individual study. The required information size (RIS) from TSA was 129, and the accrued sample size exceeded this threshold. A subgroup analysis was conducted to assess the effect of PEEP with or without a recruitment maneuver. The results showed that PEEP without a recruitment maneuver significantly reduced the risk of PPCs (OR 0.25, 95%CI: 0.13–0.49, *p* < 0.0001, *I*^2^ = 0%) (Supplementary Figure 1), and PEEP with a recruitment maneuver also reduced PPCs (OR 0.42, 95%CI: 0.18–0.99, *p* = 0.05, I^2^ = 41%) (Supplementary Figure 2).

### 3.4 Pulmonary infection

**Figure 4:**
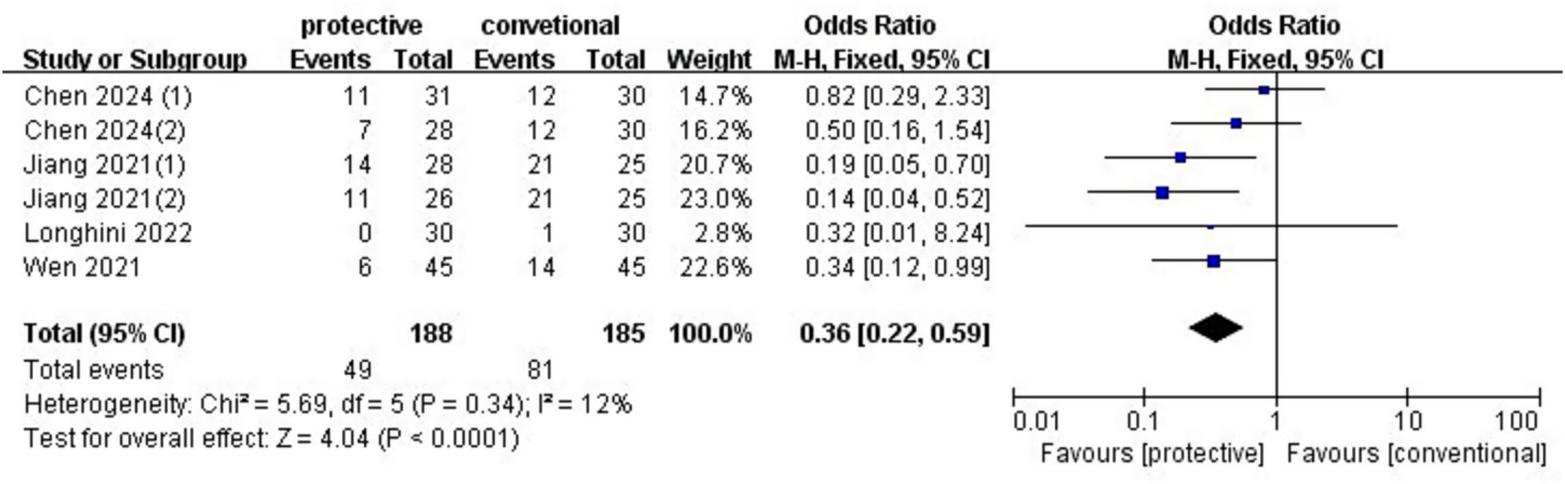
Forest plot of pulmonary infection comparing protective lung ventilation and conventional ventilation.

Four studies (*n* = 318) (4, 7–9) demonstrated that protective ventilation significantly reduced the risk of pulmonary infection (OR: 0.36, 95%CI: 0.22–0.59, *p* < 0.0001, *I*^2^ = 12%) (Figure 4). No publication bias was evident in the funnel plot, and sensitivity analysis revealed no change in the results when omitting any individual study. The RIS from TSA was 287 patients, and the accrued sample size exceeded this threshold. A subgroup analysis was conducted to assess the effect of PEEP with or without a recruitment maneuver. PEEP without a recruitment maneuver significantly reduced the risk of pulmonary infection (OR 0.27, 95%CI: 0.12–0.61, *p* = 0.002, *I*^2^ = 0%) (Supplementary Figure 3), and PEEP with a recruitment maneuver also reduced pulmonary infection (OR 0.16, 95%CI: 0.05–0.54, *p* = 0.003, *I*^2^ = 0%) (Supplementary Figure 4).

### 3.5 Atelectasis

**Figure 5:**
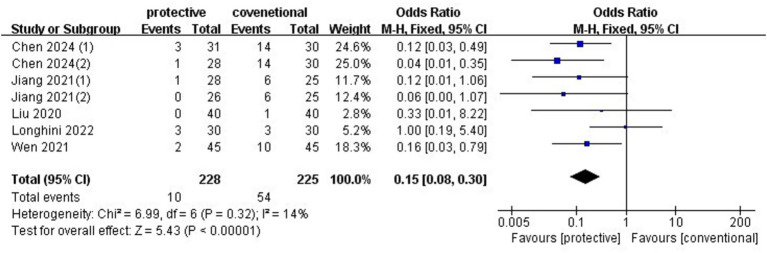
Forest plot of atelectasis comparing protective lung ventilation and conventional ventilation.

Five studies (*n* = 398) (4, 5, 7–9) showed a reduction in atelectasis with protective ventilation (OR: 0.15, 95%CI: 0.08–0.30, *p* < 0.00001, *I*^2^ = 14%) (Figure 5). No publication bias was evident in the funnel plot, and sensitivity analysis confirmed the robustness of the findings when omitting any individual study. The RIS from TSA was 185, and the accrued sample size exceeded this threshold. A subgroup analysis was conducted to assess the effect of PEEP with or without a recruitment maneuver. PEEP without a recruitment maneuver significantly reduced atelectasis (OR 0.13, 95%CI: 0.05–0.35, *p* < 0.0001, *I*^2^ = 0%) (Supplementary Figure 5), while PEEP without a recruitment maneuver did not significantly reduce atelectasis (OR 0.30, 95%CI: 0.02–5.52, *p* = 0.09, *I*^2^ = 67%) (Supplementary Figure 6).

### 3.6 ICP

**Figure 6:**
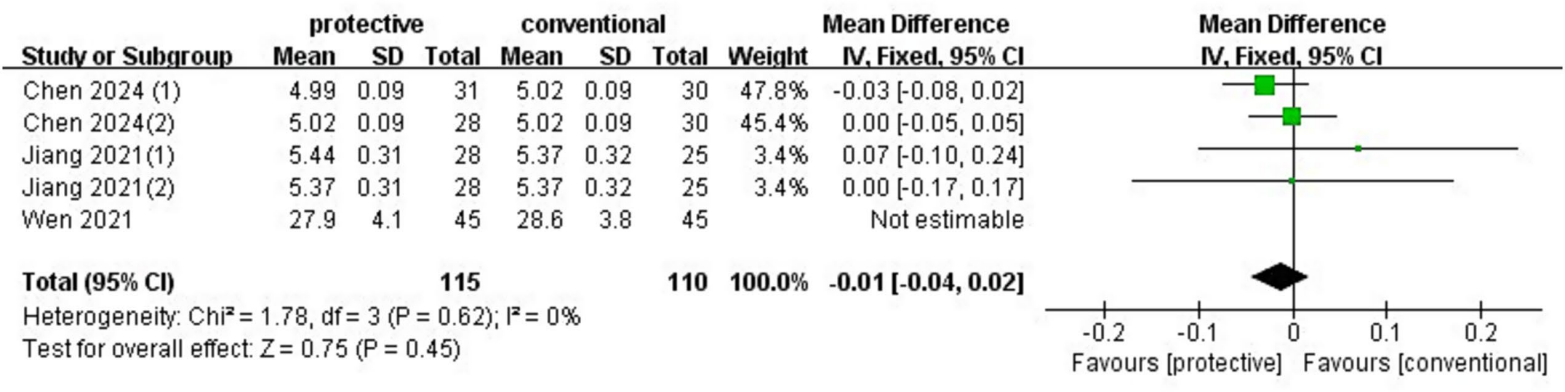
Forest plot of ONSD before dual opening comparing protective lung ventilation and conventional ventilation.

Before dual opening, one trial (7) measured ICP, and two other trials (5, 9) (*n* = 170) measured the optic nerve sheath diameter (ONSD) as a surrogate for ICP. As these measurement methods are not interchangeable, only the ONSD data were pooled for the meta-analysis. The results from the fixed-effects model showed that lung-protective ventilation did not increase ONSD (MD: −0.01, 95%CI: −0.04–0.02, *p* = 0.45, *I*^2^ = 0%) (Figure 6). The RIS from TSA was 126, and the accrued sample size exceeded the threshold.

**Figure 7:**
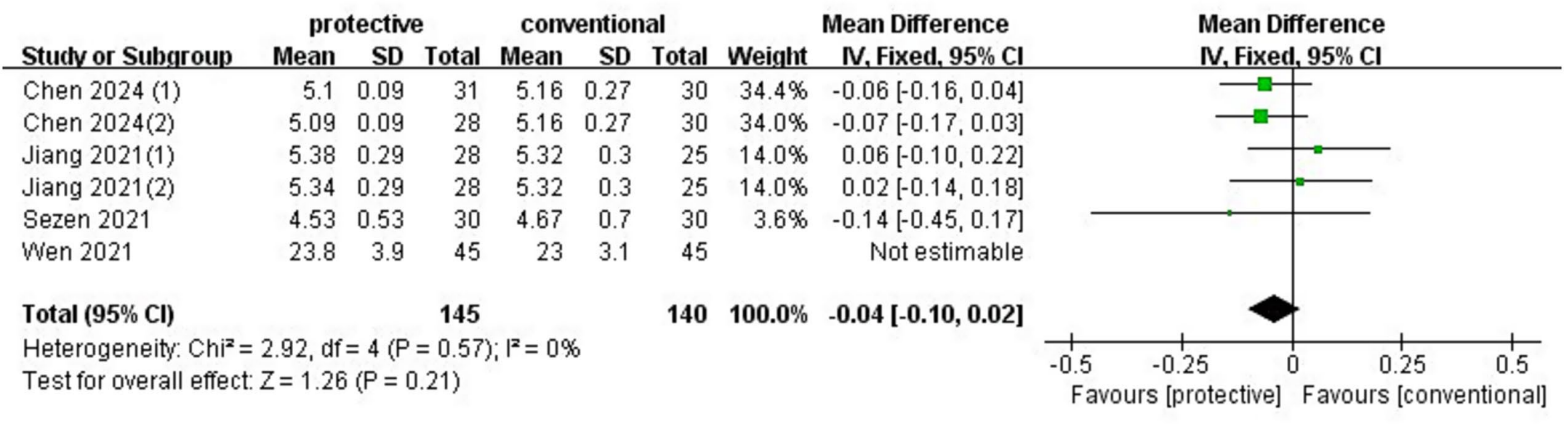
Forest plot of ONSD at the end of surgery comparing protective lung ventilation and conventional ventilation.

At the end of surgery, one trial (7) compared invasive ICP between protective lung ventilation and conventional ventilation, and three other trials (*n* = 230) (5, 9, 11) compared ONSD as a surrogate for ICP between groups. Data from these three trials reporting ONSD were pooled using a fixed-effects model, which showed no increase in ONSD with protective lung ventilation (MD: −0.04, 95%CI: −0.10–0.02, *p* = 0.21, *I*^2^ = 0%) (Figure 7). Sensitivity analysis confirmed the stability of the results when omitting any individual study. The RIS from TSA was 283, but the actual sample size in this meta-analysis was 230, which was smaller than the RIS.

### 3.7 OI

**Figure 8:**
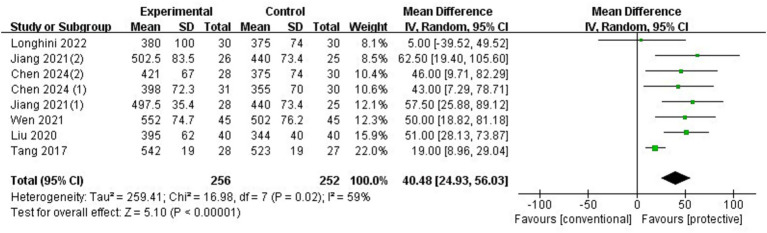
Forest plot of OI comparing protective lung ventilation and conventional ventilation.

Six trials (*n* = 453) (4, 5, 7–10) reported the oxygenation index at the end of surgery. Compared with conventional ventilation, lung-protective ventilation significantly increased the OI with an MD of 40.28 mmHg (95%CI: 24.93–56.03, *p* < 0.00001, *I*^2^ = 59%) (Figure 8). The funnel plot revealed no publication bias, and sensitivity analysis showed that the results remained unchanged even when omitting any individual trials. The RIS from TSA was 242, and the accrued sample size exceeded the threshold. The original data from the studies by Longhini et al. (4), Liu et al. (8), and Tang et al. (10) were presented as graphs, and data from the study by Jiang et al. (9) were presented as medians (interquartile range). These data were transformed into mean (SD) for this meta-analysis. Sensitivity analysis was conducted by omitting these four studies, and the results showed an MD of 46.69 mmHg (95%CI: 26.97–66.40, *p* < 0.00001, *I*^2^ = 0%), indicating robust results. A subgroup analysis was conducted to assess the effect of PEEP with or without a recruitment maneuver. PEEP without a recruitment maneuver significantly improved OI (MD 38.90 mmHg, 95%CI: 16.92–60.88, *p* = 0.0005, *I*^2^ = 66%) (Supplementary Figure 7), while PEEP with a recruitment maneuver did not significantly improve OI (MD 34.03 mmHg, 95%CI: −22.32-90.38, *p* = 0.24, *I*^2^ = 77%) (Supplementary Figure 8).

### 3.8 Lung compliance

**Figure 9:**
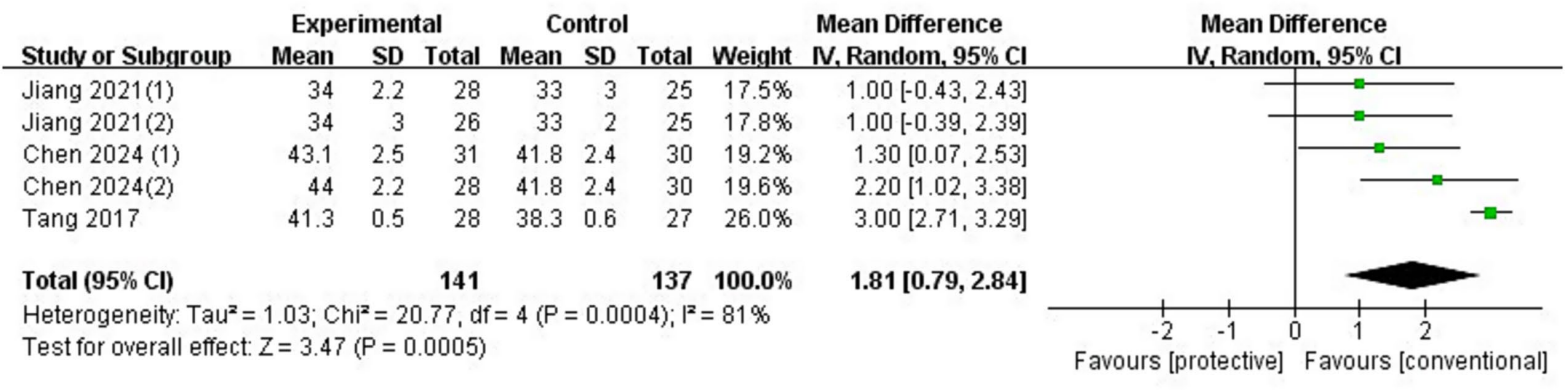
Forest plot of lung compliance comparing protective lung ventilation and conventional ventilation.

Three trials (*n* = 223) (5, 9, 10) reported lung compliance at the end of surgery, with lung-protective ventilation significantly improving OI with an MD of 1.81 mL/cmH2O (95%CI: 0.79–2.84, *p* = 0.0005, *I*^2^ = 81%) (Figure 9). Sensitivity analysis showed that the results did not change when any individual trial was omitted. The RIS from TSA was 230, but the actual sample size in this meta-analysis was smaller than the RIS.

### 3.9 Certainty of evidence from GRADE

Table 2 presents the GRADE evidence for each outcome. The quality of the evidence for overall PPCs, pulmonary infection, atelectasis, and ONSD before dual opening was moderate, while the quality of the evidence for ONSD at the end of surgery, OI, and lung compliance ranged from very low to low.

**Table 2:** Quality of evidence from GRADE.

Outcomes	Number of participants (number of studies)	OR MD 95%CI			*p*-value	*I*^2^ value	Funnel plot	Required information size (RIS)	Quality of evidence (GRADE)
		
Overall PPCs	318 (4)	0.30	∕	0.18–0.48	<0.00001	0%	Symmetrical	129	+++ (moderate)
Pulmonary infection	318 (4)	0.36	∕	0.22–0.59	<0.0001	12%	Symmetrical	287	+++ (moderate)
Atelectasis	398 (5)	0.15	∕	0.08–0.30	<0.00001	14%	Symmetrical	185	+++ (moderate)
ONSD (before dual opening)	170 (2)	∕	−0.01	−0.30–0.14	0.45	0%	∕	126	+++ (moderate)
ONSDS (end of surgery)	230 (3)	∕	−0.04	−0.105–0.02	0.21	0%	∕	283	++ (low)
OI (end of surgery)	453 (6)	∕	40.48	24.93–56.03	<0.00001	59%	Symmetrical	242	++ (low)
Lung compliance	223 (3)	∕	1.81	0.79–2.84	0.0005	81%	∕	230	+ (very low)

PPCs: postoperative pulmonary complications; ONSD: optic nerve sheath diameter; OI: oxygenation index.

## 4 Discussion

The pooled results of this meta-analysis demonstrated that intraoperative lung-protective ventilation significantly reduces the risk of postoperative pulmonary complications, improves oxygenation and lung compliance, and does not increase intracranial pressure.

PPCs are highly prevalent in neurosurgery, primarily due to prolonged intraoperative and postoperative mechanical ventilation. PEEP, a key component of LPV strategies, enhances pulmonary compliance and oxygenation while reducing atelectasis, thereby decreasing the incidence of PPCs (12). Conventional ventilation with high tidal volumes may induce localized lung inflammation and the persistent release of lung-derived cytokines (13). LPV has been shown to reduce plasma levels of proinflammatory cytokines (TNF-*α*, IL-6, and IL-8) and mitigate ventilator-induced oxidative stress associated with high oxygen concentrations (10). Consistent with these findings, our meta-analysis demonstrates that LPV significantly improves lung compliance and increases the oxygenation index, thereby reducing PPCs and supporting its clinical utility in neurosurgical procedures.

In our meta-analysis, intraoperative LPV did not elevate ICP during neurosurgical procedures. Studies examining PEEP in patients with acute brain injury in intensive care unit settings have reported inconsistent effects on ICP: some studies have observed increased ICP with elevated PEEP (14–16); others have found no significant ICP changes (17, 18); one study even reported ICP reduction during abdominal surgery (12). Given these conflicting results, the use of PEEP in neurosurgery remains cautious. Two retrospective observational studies have demonstrated that increments in PEEP (0–10 cmH_2_O) do not significantly affect ONSD during neurosurgery (19), and PEEP levels (0–8 cmH_2_O) maintain stable ICP and cerebral perfusion pressure (CPP) during neurosurgery (20, 21). One recent RCT has shown that PEEP (5 and 10 cmH_2_O) preserves regional cerebral oxygen saturation during tumor resection (22). In our meta-analysis, one included study (7) had monitored ICP using an invasive method, and three other studies had reported ONSD (5, 9, 11). We pooled the ONSD data, as ONSD serves as an indirect surrogate for ICP; the stability of ICP under LPV during the neurosurgical procedure is based on surrogate outcome measures.

This meta-analysis has some limitations. First, the definitions and timing of PPCs were inconsistent across the included studies, which may limit comparability between studies, although comparability within groups was observed in each individual study. In a recent meta-analysis, any postoperative pulmonary adverse event was considered a PPC (23). In contrast, our meta-analysis only extracted data on PPCs from original studies in which the outcome was explicitly defined as a PPC. Second, the definition of LPV varied across the included studies: the majority used fixed PEEP strategies (with or without a recruitment maneuver), while a few adopted individualized PEEP strategies. We conducted a subgroup analysis to assess the impact of PEEP with or without a recruitment maneuver on outcomes, but due to limited trial data, subgroup analyses of fixed PEEP vs. individualized PEEP, invasive ICP vs. ONSD, and elective tumor resection vs. traumatic brain injury were not feasible. Although retrospective cohort studies have suggested that high PEEP may be superior for PPC reduction (24, 25). Third, heterogeneity in OI lung compliance was observed. Although we conducted a subgroup analysis of PEEP with or without a recruitment maneuver, this heterogeneity may stem from variations in the timing of outcome measurement, patient characteristics, and ventilation strategies. Therefore, large-scale RCTs with standardized LPV protocols, consistent PPC definitions, and uniform ICP monitoring methods are needed to verify the results of our meta-analysis.

In summary, this meta-analysis demonstrates that lung-protective ventilation effectively reduces PPCs (moderate-quality evidence) and does not elevate ICP (low- to moderate-quality evidence). Given that the conclusions regarding ICP are based on surrogate measures, further large-scale RCTs with standardized measures of invasive ICP and consistent definitions of PPCs and LPV are required to validate our findings.

## Data Availability

The original contributions presented in the study are included in the article/Supplementary material, further inquiries can be directed to the corresponding author.
