# Supplementary material for: Efficacy and safety of lung-protective ventilation in neurosurgery: a systematic review and meta-analysis of randomized controlled clinical trials
Source: Front Med (Lausanne). 2026 Apr 23;13:1803798. doi: 10.3389/fmed.2026.1803798 (PMC13149440; doi:10.3389/fmed.2026.1803798)
Supplement: Supplementary file 1 [file Data_Sheet_1.docx]

PubMed:

1. positive-pressure respiration[MeSH Terms]
2. positive-pressure[All fields]
3. positive end-expiratory pressure[All fields]
4. PEEP[All fields]
5. Protective lung ventilation[All fields]
6. Protective ventilation[All fields]
7. #1 OR #2 OR #3 OR #4 OR #5 OR #6
8. Neurosurgery[MeSH]
9. Craniotomy[All fields]
10. Cerebral surgery[All fields]
11. Lobectomy[All fields]
12. Excision of brain tumor[All fields]
13. Removal of intracranial hematoma[All fields]
14. #8 OR #9 OR #10 OR #11 OR #12 OR #13
15. randomized controlled trial [ptyp]
16. randomized controlled trial as topic[MeSH Terms]
17. randomized controlled trial[All fields]
18. randomised controlled trial[All fields]
19. randomized[All fields]
20. randomized[All fields]
21. clinical trial[All fields]
22. #15 OR #16 OR #17 OR #18 OR #19 OR #20 OR #21
23. 2000/01/01 [Date-publication]: 2025/04/30 [Date-publication]
24. #7 AND #14 AND #22 AND #23

Embase

1. (‘lung protective ventilation’/exp) OR (‘positive pressure respiration’/exp) OR (‘positive end expiratory pressure’/exp)
2. (‘protective lung ventilation’ OR ‘protective ventilation’) ti ab
3. #1 OR #2
4. (‘neurosurgery’/exp) OR (‘neurosurgery’:ti ab OR ‘craniotomy’: ti ab OR ‘cerebral surgery’: ti ab)
5. ‘randomized clinical controlled trial’ OR ‘randomized controlled trial’ OR ‘clinical trial’
6. Py=2000-2025
7. #3 AND #4 AND #5 AND #6

Cochrane library

1. MeSH descriptor: [Positive-Pressure Respiration] explode all trees
2. (positive end-expiratory pressure OR protective ventilation) ti ab kw
3. #1 OR #2
4. MeSH descriptor:[neurosurgery] explode all trees
5. (craniotomy OR neurosurgery OR cerebral surgery) ti ab kw
6. #4 OR #5
7. MeSH descriptor: [Randomized Controlled Trial] explode all trees
8. (clinical trial OR randomized controlled trial) ti ab kw

9 #7 OR #8

10 Limit to 2000-2025

11. #3 AND #6 AND #9 AND #10
