# Supplementary material for: Efficacy and safety of lung-protective ventilation in neurosurgery: a systematic review and meta-analysis of randomized controlled clinical trials
Source: Front Med (Lausanne). 2026 Apr 23;13:1803798. doi: 10.3389/fmed.2026.1803798 (PMC13149440; doi:10.3389/fmed.2026.1803798)
Supplement: Supplementary file 2 [file Table_1.doc]

Supplemental Table Definitions and timings of PPCs in the included studies

| Author | PPCs definitions | timing |
| --- | --- | --- |
| Longhini etal[4] | Atelectasis, bronchospasm, hypoximia, pleural effusion, pneumonia, pneumothorax, respiratory failure | In hospitalization after surgery |
| Chen X, et al[5] | Infection, hydrothorax, atelectasis, emphysema | Within 7 days after surgery |
| Wen T, et al[6] | Atelectasis, acute lung injury, ventilator-associated pneumonia | Within 5days after surgery |
| Jiang L et al[9] | Hypoximia, pulmonary infection, atelectasis, acute respiratory distress syndrome, ventilator-associated lung injury, neurogenic pulmonary edema | Within 30 days after surgery |
