# Supplementary figures and images for: Efficacy and safety of lung-protective ventilation in neurosurgery: a systematic review and meta-analysis of randomized controlled clinical trials
Source: Front Med (Lausanne). 2026 Apr 23;13:1803798. doi: 10.3389/fmed.2026.1803798 (PMC13149440; doi:10.3389/fmed.2026.1803798)

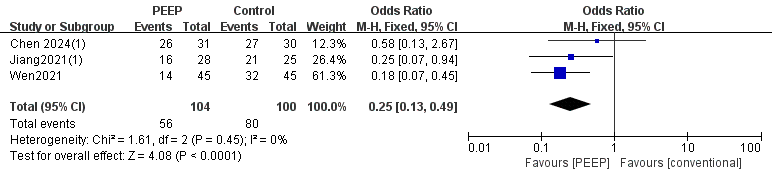

Supplement: Supplementary file 3 [file Image_1.png]

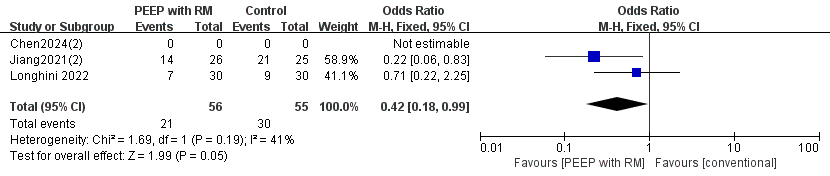

Supplement: Supplementary file 4 [file Image_2.png]

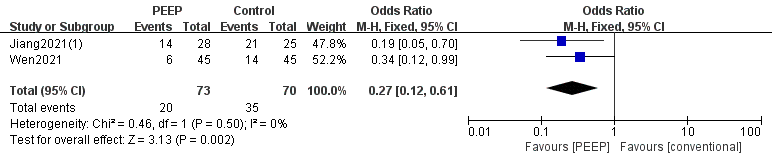

Supplement: Supplementary file 5 [file Image_3.png]

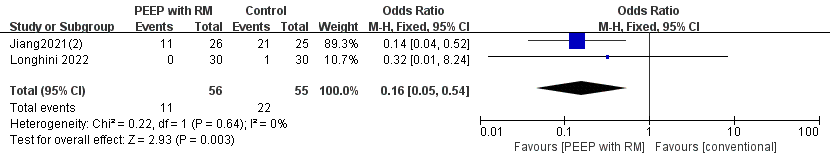

Supplement: Supplementary file 6 [file Image_4.png]

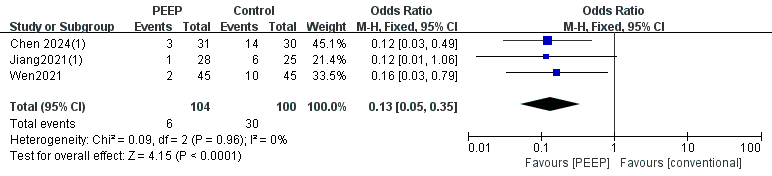

Supplement: Supplementary file 7 [file Image_5.png]

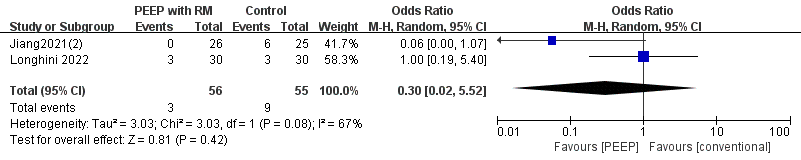

Supplement: Supplementary file 8 [file Image_6.png]

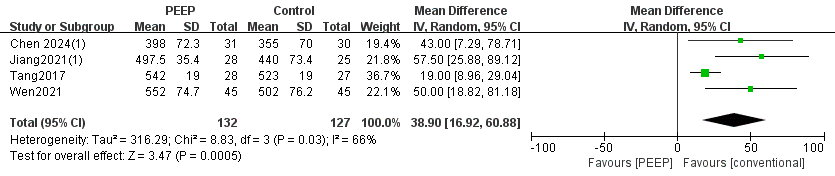

Supplement: Supplementary file 9 [file Image_7.png]

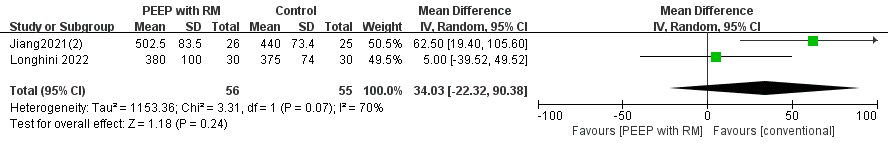

Supplement: Supplementary file 10 [file Image_8.png]
